# Assessing the validity of a Parkinson’s care evaluation: the PRIME-NL study

**DOI:** 10.1007/s10654-024-01123-7

**Published:** 2024-05-30

**Authors:** Liza M. Y. Gelissen, Robin van den Bergh, Amir H. Talebi, Angelika D. Geerlings, Bart R. Maas, Myrthe M. Burgler, Yvet Kroeze, Agnes Smink, Bastiaan R. Bloem, Marten Munneke, Yoav Ben-Shlomo, Sirwan K. L. Darweesh

**Affiliations:** 1 Radboud university medical center, Donders Institute for Brain, Cognition and Behaviour, Department of Neurology, Center of Expertise for Parkinson & Movement Disorders, Nijmegen, The Netherlands; 2 Department of Population Health Sciences, Bristol Medical School, University of Bristol, Bristol, BS8 2PS United Kingdom

**Keywords:** Parkinsonism, Parkinson’s disease, Healthcare evaluation, Healthcare model, Epidemiology, Validity, Generalizability, Selection bias, Confounding bias

## Abstract

**Introduction:**

The PRIME-NL study prospectively evaluates a new integrated and personalized care model for people with parkinsonism, including Parkinson’s disease, in a selected region (PRIME) in the Netherlands. We address the generalizability and sources of selection and confounding bias of the PRIME-NL study by examining baseline and 1-year compliance data.

**Methods:**

First, we assessed regional baseline differences between the PRIME and the usual care (UC) region using healthcare claims data of almost all people with Parkinson’s disease in the Netherlands (the source population). Second, we compared our questionnaire sample to the source population to determine generalizability. Third, we investigated sources of bias by comparing the PRIME and UC questionnaire sample on baseline characteristics and 1-year compliance.

**Results:**

Baseline characteristics were similar in the PRIME (n = 1430) and UC (n = 26,250) source populations. The combined questionnaire sample (n = 920) was somewhat younger and had a slightly longer disease duration than the combined source population. Compared to the questionnaire sample in the PRIME region, the UC questionnaire sample was slightly younger, had better cognition, had a longer disease duration, had a higher educational attainment and consumed more alcohol. 1-year compliance of the questionnaire sample was higher in the UC region (96%) than in the PRIME region (92%).

**Conclusion:**

The generalizability of the PRIME-NL study seems to be good, yet we found evidence of some selection bias. This selection bias necessitates the use of advanced statistical methods for the final evaluation of PRIME-NL, such as inverse probability weighting or propensity score matching. The PRIME-NL study provides a unique window into the validity of a large-scale care evaluation for people with a chronic disease, in this case parkinsonism.

**Supplementary Information:**

The online version contains supplementary material available at 10.1007/s10654-024-01123-7.

## Introduction

Parkinson’s disease (PD) is a neurodegenerative progressive and chronic syndrome affecting roughly 6.1 million people globally [1]. The clinical presentation and progression is highly heterogeneous, whilst current models of care insufficiently address the person-specific needs of people with PD and related neurodegenerative diseases characterized by parkinsonism [2]. Models of care for chronic, neurological disorders could specifically enhance their multidisciplinary collaboration, timely detection and proactive management of problems, and further facilitate the empowerment and involvement of people with parkinsonism and carers in their own healthcare process [3]. To address these challenges, an international panel of multidisciplinary healthcare professionals designed a new integrated and personalized care model for people with parkinsonism called ‘PRIME Parkinson’: Proactive and Integrated Management and Empowerment in Parkinson’s disease [4]. The model seeks to achieve a quadruple aim of healthcare [5, 6]: enhancing patient and carers experience of care, improving population health, maintaining neutral healthcare costs and improving professional fulfilment of healthcare providers involved in the care of people with parkinsonism.

In the Netherlands, the PRIME Parkinson care model has gradually been introduced as a replacement of usual care from 2021 onwards in one tertiary healthcare centre and four regional hospitals (PRIME region) [7]. We focused on hospital-based care as the majority of people with PD in the Netherlands (> 95%) receive it. Except for the PRIME region, the rest of the Netherlands continued providing usual care (UC region). To determine the impact of the PRIME Parkinson care model with regard to the quadruple aim, a prospective multifaceted evaluation was initiated called the PRIME-NL study. Note that a complementary study is underway in the south-west of England, termed the PRIME-UK study [8].

The PRIME-NL study collects both healthcare claims data and annual questionnaires in the PRIME and UC region for five years. We use the healthcare claims data to assess the population health domain of the quadruple aim by measuring, amongst other variables, the occurrence of parkinsonism-related complications amongst all people with Parkinson’s disease (PD) in the Netherlands. The annual questionnaires include a questionnaire sample of people with parkinsonism, care partners and healthcare professionals from both the PRIME and UC region. The questionnaires cover a broad range of topics addressing the four domains of the quadruple aim, such as experience of care, quality of life, empowerment and healthcare professional fulfilment. Data collection for PRIME-NL started in January 2020, one year before the implementation of the PRIME Parkinson care model, which serves as the baseline measurement. In this paper, we only analyse the data from persons with parkinsonism because we also had access to their healthcare claims data, unlike the situation for carers and healthcare professionals.

**Fig. 1:**
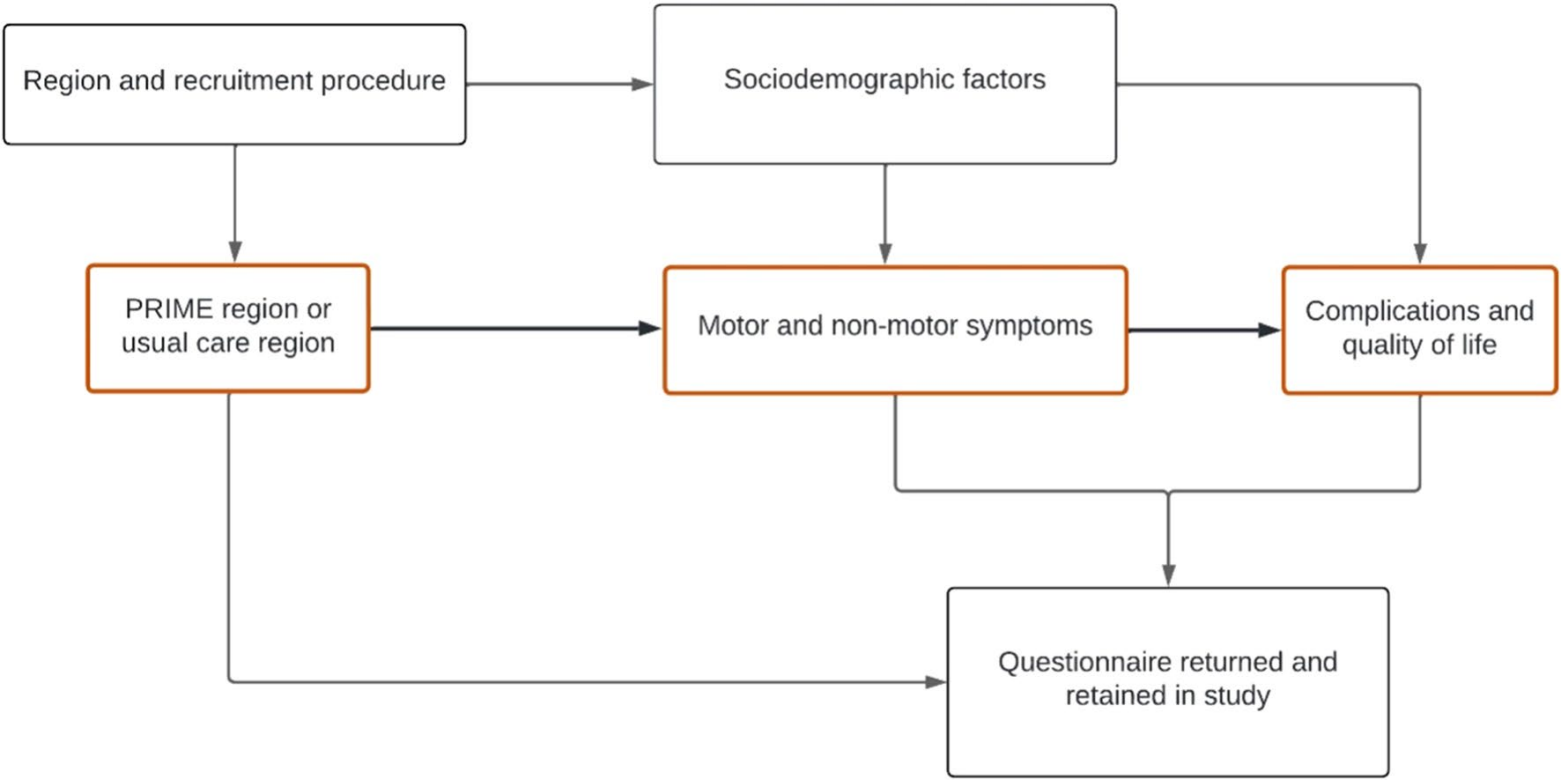
Directed Acyclic Graph (DAG) of possible potential sources of bias that may influence the eventual evaluation of the PRIME-NL study. In orange, a highly simplified version of the effect of PRIME care is displayed (middle pathway): improved care can ameliorate motor and non-motor symptoms which in turn reduce the amount of complications and improve the quality of life. However, for an adequate evaluation of the PRIME Parkinson care model, several methodological challenges and potential sources of bias need to be identified. First, PRIME Parkinson care has been implemented in a specific, non-randomized region of the Netherlands which might be different from the rest of the Netherlands (UC region) at baseline. The regions can differ in sociodemographic factors that impact the presence of symptoms, complications, and quality of life (top pathway). Sociodemographic factors can thereby introduce confounding bias, e.g., the PRIME participants are older, and older age is associated with more symptoms and more complications, making PRIME look worse on the final evaluation when not correcting for age. Second, we might have differentially recruited people from the source populations into the questionnaire sample, e.g., through the letter by the neurologists in the PRIME region. This letter might have reached specific subgroups of participants in the PRIME region, e.g., people with more symptoms, introducing selection bias. Third, collider bias might create an artificial association between the region and outcomes when differential loss to follow-up occurs. For example, if we assume that we have recruited more affected people in the PRIME region and participants with more symptoms are less likely to return their questionnaire, the PRIME region will appear worse compared to UC in which fewer highly affected participants are retained (bottom pathway). We have not illustrated information bias in this DAG since participants were unaware of the study group at baseline. However, at follow-up, because the study is unblinded, they will be aware and this could introduce differential measurement error

Because of the real-life nature of the evaluation, several methodological challenges may hamper a valid evaluation of the PRIME Parkinson care model. Three questions stand out in particular and will be addressed in this paper. The first question is whether the source population of people with PD differs between the PRIME and UC region. The second question is whether the combined questionnaire sample of participants from both regions is representative of all people with PD in the Netherlands, i.e., whether the questionnaire sample findings can be generalized to the source population. The third question is whether potential selection and confounding bias is the same or different between the PRIME and UC questionnaire sample, i.e., we examine those as important aspects of the internal validity. Figure 1 demonstrates the conceptual framework of possible pathways through which selection and confounding bias may affect the evaluation of PRIME-NL.

## Methods

### Overview

**Fig. 2:**
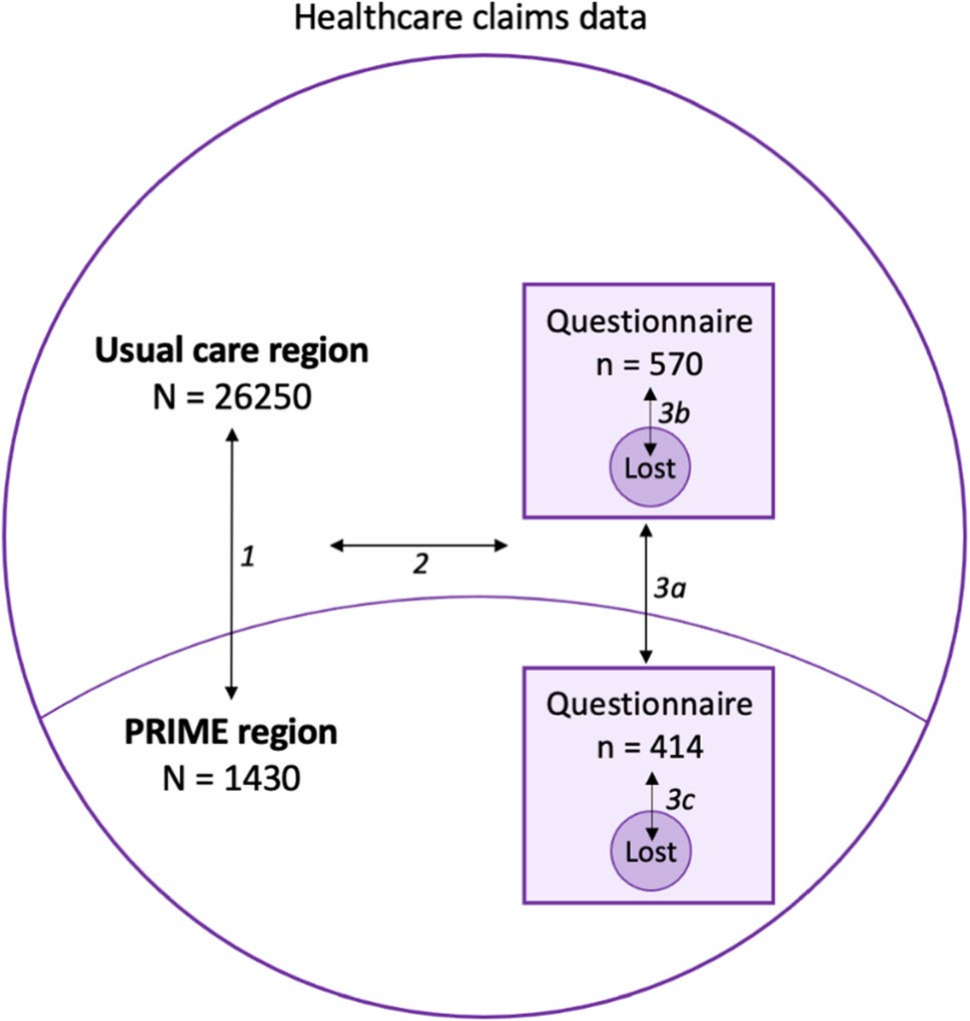
Overview of the comparisons made. First, we assessed regional baseline differences between the PRIME and the usual care (UC) region using healthcare claims data of almost all people with Parkinson’s disease in the Netherlands (the source population) (1). Second, we compared the combined questionnaire sample of participants from both regions to the source population to determine if the questionnaire sample findings will be generalizable to all people with PD in the Netherlands (2). Third, to assess selection and confounding bias between the two regions, we compared the PRIME and UC questionnaire sample on baseline characteristics (3a) and investigated whether there is differential 1-year compliance (3b and 3c)

The source population is defined as all people with PD in the Netherlands, divided in either the PRIME or the usual care region receiving hospital-based neurological care [7]. From both source populations, we recruited a questionnaire sample containing an unmatched and self-enrolled group of people with PD (convenience sampling). To examine the research questions, we 1) investigated the regional differences in baseline characteristics between the source population in the PRIME and UC region in the healthcare claims data, 2) determined the generalizability of the questionnaire sample by comparing their characteristics to the source population, and 3) tested for the presence of selection and confounding bias by comparing the PRIME and UC questionnaire sample at baseline and 1-year follow-up (Fig. 2).

### Healthcare claims data on source population

People with PD were identified in the national healthcare claims data of Vektis, which contains the data of more than 99% of all people with PD in the Netherlands. For this specific analysis, we included only people with PD, based on diagnostic hospital code DBC501, because the diagnostic hospital code for atypical parkinsonism is also used for other types of movement disorders. The inclusion criteria were: 1) received the 501 code in 2018, 2019 or 2020, 2) alive in 2020, and 3) primarily received outpatient care at a regional hospital instead of a university medical centre, because PRIME Parkinson care is restricted to regional hospitals as they better reflect usual care for the majority of people with parkinsonism. The hospital of care was classified as regional if people with PD received more than 75% of their care in a regional hospital in the years 2018, 2019 and 2020. In our analysis of baseline data, we examined regional differences in age, sex, disease duration, socio-economic status, Charlson comorbidity index, hospital admissions for orthopaedic fractures and pneumonia’s, as well as prescribed medication for anxiety, depression, and cognitive impairments.

Furthermore, we leveraged data from the Central Bureau of Statistics (CBS) of the Netherlands to determine regional differences regarding variables not included in the healthcare claims database [9]. This includes migratory background, overweight based on body mass index (BMI), COVID-19 occurrence, smoking behaviour, alcohol consumption, education, and living situation. Although these data are extracted from the general population instead of the PD specific population, they are the only and best proxy for determining regional differences at baseline for the PD population for the variables missing in the healthcare claims data. If a relationship between these variables and PD exists [10], we assumed that such a relationship will be similar between both regions. We extracted data on a provincial level because no municipality-level data were available (see supplementary file S1 for details). Therefore, in this analysis only, we used the provinces Gelderland, Noord-Brabant and Limburg as a proxy for the PRIME region, because they cover the population of the PRIME hospitals [7]. We are mindful that these provinces also include considerable subregions that are not part of the PRIME region, so we interpret this analysis with caution.

### Questionnaire sample

#### Participants

People with a clinical diagnosis of parkinsonism, which was confirmed by a letter of the general practitioner or neurologist, were eligible to participate in the questionnaire study, irrespective of whether the specific diagnosis was PD or atypical parkinsonism. People with medication-induced parkinsonism and those who received their treatment in university medical centres were excluded. Potential participants must have visited the neurology outpatient clinic of a regional hospital at least once during the last year for inclusion in questionnaire-based assessments [7].

#### Materials

The questionnaire consisted of various tailor-made sub-questionnaires aimed at retrieving socio-demographic characteristics as well as several existing (clinical) questionnaires to measure, e.g., depression or anxiety. For this paper, the following variables were examined: region, recruitment procedure, sex, age, disease duration, COVID-19 burden, education, work situation, living situation, smoking behaviour, alcohol consumption, BMI, comorbidities, anxiety, depression, cognition, complications, motor symptoms, disease stage based on the Hoehn and Yahr score, and quality of life. All items in the questionnaire were mandatory to complete for participants. An overview of included variables and associated questionnaires is provided in Supplementary Table 1.

#### Procedures

Prior to study inclusion, potential participants were called by one of the well-trained research assistants of the assessment team to inform them about study procedures and screen on inclusion criteria. When eligible for the study, participants were sent an informed consent form. Participants had up to 10 days to think about participating in the study. They were called again to discuss any questions and, if they were still interested, to sign the informed consent and to assess cognitive performance using the telephone Montreal Cognitive Assessment (t-MoCA). Afterwards, participants could either self-complete questionnaires electronically or on paper or answer the questions during a phone call with one of the research assistants. Only the paper version of the questionnaire allowed participants to not complete questions. If this was the case, the assessment team called, e-mailed or sent a letter to the participant to complete the questionnaire(s). When the questionnaire was administered via a phone call, the research assistant would encourage the participant to answer all questions.

We implemented identical recruitment strategies in both regions, except for an additional information letter sent by the treating neurologists to the persons with parkinsonism in the PRIME region because recruitment was lagging behind (Table 1).

**Table 1:** Recruitment procedures and strategies to restrain the loss to follow-up in the PRIME-NL study

Recruitment procedures (adapted from [7])		
Phase 1	PRIME region and UC region	Invitation letters were sent to members of ParkinsonNEXT across the Netherlands^a^
		The Parkinson Association^b^ sent newsletters to their members and shared posts on their website
		A brochure with a reply card was shared with potential participants at different events for people with parkinsonism and their carers
	Exclusively in the PRIME region	Neurologists sent information letters to all people with parkinsonism they treated
Phase 2	PRIME region and UC region	People with parkinsonism who were interested in participating could express this on the website or by contacting the assessment team via telephone, email or sending a reply card by post. Subsequently, they were provided with more information about the study by a call from a member of the research team
Phase 3	PRIME region and UC region	People with continued interest in participating received an information letter and consent form by e-mail or post, to let the people with parkinsonism sign the informed consent form
*Efforts of the assessment team to encourage people with parkinsonism for participation*		
1	The assessment team analysed through sampling in the questionnaire sample how participants want to be informed about the study, and how they wish to be involved in study	
2	Participants are called personally, as much as possible by the same assessor, prior to each questionnaire to inform them that the questionnaire is coming up again. This call also includes a brief re-iteration of the study content and participants can ask questions	
3	Every year, in December, a personal Christmas card is sent to every participant	
4	A quarterly newsletter is sent out in which the latest updates of the study are shared with the participants	
5	During office hours, the assessors were available for participants in case of unclarity or questions through telephone and email	

^a^A web-based platform for people with parkinsonism and their carers who have expressed an interest in participating in research

^b^A Dutch association for people with Parkinson’s disease and parkinsonism

### Statistical analyses

#### Source population differences

The healthcare claims and CBS data were used to examine the regional demographic differences at baseline (2020) between persons with PD in the PRIME and UC region (Table 2A). We used t-tests for age, disease duration, socioeconomic status and comorbidities. For each outcome, we inspected histograms and standard deviations per group to assess the assumptions of normality and homoscedasticity. If these assumptions were violated, we performed the Mann–Whitney U-test instead of the t-test. We performed Chi-square tests for sex, anti-anxiety medication, anti-depressive or cognitive medication, orthopedic fractures and pneumonia’s to compare both regions. For the CBS data comparisons (Table 2B), we performed no statistical tests as these data reflect population-measures. We adhered to a 5% difference as cut-off for meaningful differences.

**Table 2:** Comparison of baseline characteristics in A) the UC and PRIME source populations based on the healthcare claims data, B) the same comparison based on the CBS data and C) the source population as a whole and the PRIME-NL questionnaire sample

A	UC region – claims data (n = 26,250)	PRIME region – claims data (n = 1430)	Mean difference or odds ratio [95% CI]^b^	p
Age: mean (SD)^a^	72.7 (9.1) (men 72.1 (9.1); women 73.6 (9.2))	73.0 (8.9) (men 72.4 (8.7); women 73.9 (9.1)	0.3 [-0.18, 0.78]	.22
Sex (men): n (%)	15,794 (60)	872 (61)	1.04 [0.93, 1.15]	.54
Disease duration in years: mean (SD)^c^	5.3 (3.5)	5.1 (3.5)	0.2 [0.01, 0.39]	< .0001
Participants with at least one anti-depressive or cognitive medication: n (%)	6621 (25)	320 (22)	0.85 [0.75, 0.97]	.016
Participants with at least one anti-anxiety medication: n (%)	3906 (15)	170 (12)	0.77 [0.66, 0.91]	.002
Charlson comorbidity index: mean (SD)	2.91 (1.00)	2.95 (0.99)	0.04 [-0.01, 0.09]	.14
Socioeconomic status (standardized): mean (SD)	-0.11 (1.1)	-0.25 (1.1)	-0.14 [-0.20, -0.08]	< .0001
Participants with at least one fracture: n (%)	940 (3.6)	56 (3.9)	1.10 [0.83, 1.45]	.51
Participants with at least one pneumonia: n (%)	359 (1.4)	18 (1.3)	0.92 [0.57, 1.48]	.73

B	UC region – CBS data	PRIME region – CBS data
Migratory background: n (%)^d^	146,895 (5.5)	107,912 (7.0)
Overweight: n (%)	1,147,860 (56.9)	683,477 (58.2)
COVID-19 hospitalizations: n (%)	13,805 (0.61)	10,140 (0.80)
Smoking: n (%)	190,114 (9.4)	107,498 (9.2)
Excessive alcohol consumption: n (%)	154,825 (7.7)	80,192 (6.8)
*Education: n (%)*^*e*^		
Primary	2,517,444 (27.6)	1,438,755 (31.1)
Secondary	3,657,181 (40.0)	1,870,024 (40.5)
Tertiary	2,960,875 (32.4)	1,311,749 (28.4)
*Living situation: n (%)*		
Alone	849,094 (44.1)	416,979 (40.5)
With partner or child(ren)	1,074,807 (55.9)	612,565 (59.5)

C	Source population – claims data (n = 27,680)	Questionnaire sample – questionnaire data (n = 920)^f^	Mean difference or odds ratio [95% CI] ^b^	p
Age: mean (SD)	72.7 (9.1)	68.4 (8.0)	-4.30 [-4.90, -3.71]	< .0001
Sex (men): n (%)	16,666 (60)	536 (58)	0.92 [0.81, 1.05]	.23
Disease duration in years: mean (SD)	5.3 (3.5)	6.65 (5.3)	1.35 [1.12, 1.59]	< .0001
Participants with at least one pneumonia: n (%)	377 (1.4)	23 (2.5)	1.86 [1.21, 2.84]	.004

^a^SD = standard deviation.

^b^CI = confidence interval. T-tests were applied on age, the Charlson comorbidity index and socioeconomic status; a Mann-Whitney U-test was used for disease duration due to non-normal distributions in both groups for Table 2A, for disease duration in Table 2C we used a t-test because of the inverse probability weighting; Chi-square tests for independence were applied on sex, participants with at least one anti-depressive or cognitive medication, participants with at least one anti-anxiety medication, participants with at least one fracture and participants with at least one pneumonia.

^c^Disease duration was determined by the number of years from first 501 code

^d^CBS data for this outcome is based on people aged > 60 years, other variables are based on people > 65 years

^e^CBS data for this outcome is based on people aged > 18 years, due to changes in the educational system no data was available on a provincial level for only people > 60 years

^f^We included only people with PD and applied inverse probability weighting based on the sampling ratio to account for selective overrepresentation of the PRIME region participants

#### Generalizability

We tested whether the source population and questionnaire sample, both with combined regions, were different in age and disease duration with t-tests. For sex and the number of pneumonia’s, we performed Chi-square tests to compare the source population and questionnaire sample. To make a fair comparison to the source population, we excluded the people with atypical parkinsonism from the questionnaire sample for this analysis. Furthermore, we adjusted the combined questionnaire sample estimates through inverse probability weighting. This was necessary to account for the selective overrepresentation of PRIME participants in the questionnaire sample, as we recruited 27% of the PRIME source population versus 2% of the UC source population.

#### Selection and confounding bias

To examine the presence of selection bias and the potential for confounding bias in the questionnaire sample, we tested whether the PRIME region and the UC region (predictor) differed with respect to baseline characteristics (outcome). Furthermore, to assess whether the recruitment procedure introduced selection bias, we compared people within the PRIME region who were recruited by their neurologist with people who were not recruited by their neurologist (predictor) on baseline characteristics (outcome). For both analyses, we used linear regression for continuous outcomes and multinomial or binary logistic regression for nominal and ordinal outcomes, adjusting all analyses for age, sex and disease duration. Outliers were included. Continuous variables that did not meet the assumptions for linear regression were log transformed before conducting linear regression.

To examine if the loss to follow-up caused selection bias, differences between participants who remained in the study and who were lost were assessed with linear regression for continuous outcomes (age, motor symptoms, depression, anxiety, cognition, quality of life, disease duration) and with multinomial (education and disease stage) or binomial (sex) logistic regression, using compliance as predictor in all models. We performed these analyses for each region separately as we expect a test for interaction across all outcomes and regions to be underpowered given the low number of drop-outs. We log-transformed continuous outcomes that did not meet the assumptions for linear regression. We define a loss to follow-up as a participant who no longer provided questionnaire data for any reason. Therefore, the loss to follow-up numbers contain both deceased participants as well as actively dropped-out participants. All p-values were adjusted according to the Benjamini–Hochberg method [11].

All data analyses were conducted in R Studio version 2022.02.1 [12]. We https://osf.io/wugkc/?view_only=5f8d8725072a46deb6fcc2ce77fb7881 pre-registered our analyses at the Open Science Framework. In our interpretation of all analyses, we consider both p-values, effect estimates and confidence intervals to judge whether differences between groups are meaningful.

## Results

### Source population differences and generalizability

Based on the inclusion criteria, data from 27,680 people with PD were extracted from the healthcare claims data. The source populations of people with PD were similar in both regions in terms of age, sex, comorbidity scores, and number of fractures and pneumonias (Table 2A). However, people with PD living in the PRIME region had a slightly shorter disease duration (0.2 years, 95% confidence interval (CI) 0.01–0.39, *p* < .0001), used fewer anti-depressive or cognitive medications (odds ratio (OR) 0.85, 95% CI 0.75–0.97, *p* = .016), used fewer anti-anxiety medications (OR 0.77, 95% CI 0.66–0.91, *p* = .002) and had a lower socioeconomic status (mean difference = −0.14, 95% CI −0.20 to −0.08, *p* < .0001) compared to people with PD in the UC region. The CBS data showed no meaningful differences between the PRIME and UC source populations (Table 2B).

People with PD in the questionnaire sample were younger than the source population (−4.30 years, 95% CI −4.90 to −3.71, *p* < .0001), had a longer disease duration (1.35 year, 95% CI 1.12–1.59, *p* < .0001) and experienced more pneumonia’s (OR 1.86, 95% CI 1.21–2.84, *p* = .004). The proportion of men and women was similar in the questionnaire sample and source population (Table 2C).

### Selection and confounding bias in the questionnaire sample

#### Differences in baseline characteristics

In total, 984 participants completed the baseline questionnaire, including 920 people with PD (93.5%) and 64 people with atypical parkinsonism (6.5%). In both the PRIME and the UC region, most participants answered the questionnaire online (54% and 78% respectively). However in the PRIME region more people filled in the paper questionnaire (45%) compared to the UC region (21%; supplementary Table 2). Table 3 presents an overview of all baseline characteristics and their distribution across both regions. Compared to the questionnaire participants in the UC region, the participants in the PRIME region were older and had more cognitive impairments. The participants in the UC region had a longer disease duration than the PRIME participants, were more likely to receive tertiary education and tended to drink alcohol more often. No statistically significant differences were found between the PRIME and UC participants on the other outcomes when correcting for differences in age, sex and disease duration. However, the region-specific estimates suggest that participants in the PRIME region may have had more anxiety, a slightly higher BMI and a lower quality of life than participants in the UC region.

**Table 3:** Baseline characteristics of questionnaire participants

Variables^a^		Overall (n = 984)	PRIME (n = 414)	Usual Care (n = 570)	(Log−)B−weights / odds ratio [95% CI]^b^	Adj. *p*
Age (years): mean (SD)		69.7 (8.1)	71.8 (7.8)	68.2 (7.9)	-3.54 [-4.53, -2.55]	<.0001
Sex (men): n (%)		601 (61)	271 (65)	330 (58)	0.83 [0.63, 1.08]	.43
Diagnosis PD: n (%)		920 (95)	381 (92)	539 (95)	1.30 [0.76, 2.20]	.49
Disease duration (years): mean (SD)^c^		6.20 (5.2)	5.68 (5.2)	6.58 (5.3)	0.18 [0.09, 0.26]	.001
Migratory background: n (%)		9 (1)	6 (1.5)	3 (0.5)	0.42 [0.09, 1.72]	.45
BMI: mean (SD)		25.6 (4.0)	25.9 (4.2)	25.4 (3.9)	-0.68 [-1.20,-0.16]	.06
Motor symptoms: mean (SD)		12.5 (7.8)	13.0 (8.2)	12.2 (7.5)	-0.53 [-1.43, 0.38]	.45
Depression: mean (SD)		11.8 (6.9)	12.1 (7.0)	11.6 (6.8)	-0.61 [-1.50, 0.29]	.43
Anxiety: mean (SD)		38.0 (9.4)	38.9 (9.5)	37.4 (9.3)	-1.63 [-2.85, -0.41]	.06
Cognition: mean (SD)^d^		18.0 (3.0)	17.2 (3.1)	18.6 (2.7)	0.06 [0.04, 0.09]	<.0001
Quality of life: mean (SD)		73.9 (13.2)	73.0 (13.3)	74.6 (13.0)	1.95 [0.33, 3.57]	.09
COVID−19 burden: mean (SD)		2.50 (0.89)	2.44 (0.92)	2.56 (0.87)	0.14 [0.01, 0.26]	.16
*Disease stage*: *n* *(%)*^*e*^						
Stage 1		282 (29)	115 (28)	167 (29)	Reference	
Stage 2		336 (34)	134 (32)	202 (35)	1.11 [0.79, 1.55]	.67
Stage 3		150 (15)	64 (16)	86 (15)	0.99 [0.64, 1.52]	.99
Stage 4		156 (16)	70 (17)	86 (15)	1.01 [0.64, 1.57]	.99
Stage 5		40 (4)	23 (6)	17 (3)	0.65 [0.32, 1.34]	.45
*Comorbidities*: *n* *(%)*^*f*^						
Cardiovascular disease		224 (23)	108 (26)	116 (20)	0.89 [0.65, 1.22]	.61
Pulmonary disease		94 (10)	45 (11)	49 (9)	0.80 [0.51, 1.23]	.49
Musculoskeletal disorder		286 (29)	124 (30)	162 (28)	0.94 [0.70, 1.27]	.79
Endocrine or metabolic disorder		95 (10)	47 (11)	48 (8)	0.80 [0.53, 1.30]	.49
Neuropsychiatric disorder		87 (9)	44 (11)	43 (8)	0.66 [0.42, 1.05]	.27
Cancer		86 (9)	48 (12)	38 (7)	0.70 [0.44, 1.12]	.42
No comorbidity		406 (41)	150 (36)	256 (45)	1.31 [1.00, 1.72]	.19
*Smoking: n (%) *						
Smoked in the past		544 (55)	238 (57)	306 (54)	Reference	
Never smoked		411 (42)	160 (39)	251 (44)	1.00 [0.76, 1.31]	.99
Current smoker		29 (3)	16 (4)	13 (2)	0.46 [0.57, 1.02]	.21
*Alcohol consumption*: *n* *(%) *						
Never		193 (20)	99 (24)	94 (17)	Reference	
Very rarely		193 (20)	87 (21)	106 (19)	1.32 [0.87, 2.01]	.45
Occasionally		250 (25)	112 (27)	138 (24)	1.35 [0.90, 1.99]	.42
Less than 5 days a week		167 (17)	63 (15)	104 (18)	1.80 [1.16, 2.83]	.06
5 or more days a week		181 (18)	53 (13)	128 (22)	3.46 [2.18, 5.42]	<.0001
*Education*: *n (%) *						
Primary		252 (25)	154 (38)	98 (11)	Reference	
Secondary		258 (26)	114 (27)	144 (25)	1.86 [1.28, 2.72]	.008
Tertiary		474 (48)	146 (35)	328 (58)	3.90 [2.77, 5.47]	<.0001
*Working situation*: *n* *(%) *						
Retired		681 (69)	316 (76)	365 (64)	Reference	
Fulltime		26 (3)	12 (3)	14 (2)	0.57 [0.20, 1.36]	.43
Parttime		54 (5)	21 (5)	33 (6)	0.60 [0.28, 1.25]	.43
Self−employed		36 (4)	15 (4)	21 (3)	0.69 [0.34, 1.52]	.54
Incapacitated and/or receiving sickness benefit		151 (15)	38 (9)	113 (20)	0.94 [0.61, 1.99]	.80
Unemployed		25 (3)	10 (2)	15 (3)	0.59 [0.23, 1.46]	.45
Voluntary work or following education not paid by employer		11 (1)	2 (1)	9 (2)	2.69 [0.52, 12.06]	.45
*Living situation: n (%)*						
With partner		755 (77)	321 (77)	434 (76)	Reference	
Alone		137 (14)	62 (15)	75 (13)	0.82 [0.55, 1.20]	.49
With partner and children		65 (6)	19 (5)	46 (8)	0.97 [0.51, 1.86]	.99
In an institution, assisted living or sheltered living		19 (2)	8 (2)	11 (2)	1.41 [0.52, 3.78]	.63
Living with another family member than partner		8 (1)	4 (1)	4 (1)	0.44 [0.10, 1.88]	.45
*Complications in the year prior to the first questionnaire–Urinary tract infection, pneumonia, falling, hallucinations: n (%) *						
Any complication reported		505 (51)	220 (53)	285 (50)	0.89 [0.67, 1.16]	.53
*Specific complications–not reported/ reported/ led to hospital admission: n (%) *						
Urinary tract infection	Not reported	891 (91)	374 (90)	517 (91)	Reference	
	Reported	78 (8)	34 (8)	44 (8)	0.79 [0.47, 1.31]	.51
	Hospitalized	15 (1)	6 (2)	9 (1)	1.25 [0.41, 3.74]	.79
Pneumonia	Not reported	948 (97)	393 (95)	555 (97)	Reference	
	Reported	23 (2)	12 (3)	11 (2)	0.74 [0.32, 1.75]	.64
	Hospitalized	13 (1)	9 (2)	4 (1)	0.41 [0.12, 1.36]	.42
Falling	Not reported	561 (57)	230 (56)	331 (58)	Reference	
	Reported	399 (41)	172 (41)	227 (40)	0.93 [0.70, 1.23]	.72
	Hospitalized	24 (2)	12 (3)	12 (2)	0.61 [0.26, 1.45]	.45
Hallucinations	Not reported	858 (87)	357 (86)	501 (88)	0.13 [0.76, 1.70]	.66
	Reported	126 (13)	57 (14)	69 (12)		

^a^SD = Standard Deviation; BMI = Body Mass Index. Motor symptoms*:* Movement Disorders Society Unified Parkinson Disease Rating Scale (MDS-UPDRS) Part II, ranging from 0 to 52, higher score indicates a greater degree of motor symptoms. Depression: Beck Depression Inventory II (BDI), ranging from 0 to 63, higher scores indicate greater depressive severity. Anxiety: State Trait Anxiety Inventory for Adults (STAI); only the Trait Anxiety Scale was included, ranging from 20 to 80, higher score indicates a greater degree of anxiety. Cognition: Telephone Montreal Cognitive Assessment (t-MoCA), ranging from 0 to 22, higher score indicates better cognitive performance. Quality of life: Parkinson’s Disease Questionnaire-39 (PDQ-39), ranging from 0 to 100, higher score indicates a better quality of life. COVID-19 burden: COVID-19 questionnaire containing 8 questions, the average is calculated and ranges from 0 to 5, higher score indicates a higher COVID-19 burden. Disease duration: Years since diagnosis of PD or parkinsonism. Disease stage: Hoehn & Yahr scale, this score was calculated on answers from other questionnaires, notably the UPDRS. Scores can range from 1 to 5 (disease stage 1 to 5) in which a higher stage indicates more severe disease. Education: Primary educated = no education, primary school, VMBO (see also Table S1); Secondary educated = HAVO, VWO, MBO; Tertiary educated = HBO, University, PhD. Hospitalized: Complication led to a hospital admission

^b^CI = confidence interval; all continuous variables were analyzed using linear regression, all binary variables were analyzed using binomial logistic regression, all categorial variables with more than two categories, including ordinal variables, were analyzed using multinomial logistic regression. Continuous variables that did not meet the assumptions for linear regression were log transformed before conducting linear regression. All tests between PRIME and UC were adjusted for age, sex and disease duration, excluding the tests for age, sex and disease duration. We reported odds ratios for all variables tested with binomial or multinomial logistic regression, log-b-weights for all log transformed variables tested with linear regression, and b-weights for all other variables tested with linear regression

^c,d^Log transformed before tested with linear regression

^e^Not 100% in total due to NAs

^f^Not 100% in total since participants could have comorbidities in more than one category

#### Impact of recruitment strategy

In the PRIME region, 263 participants (66%) indicated that they were introduced to the PRIME-NL study by their neurologist. Although not statistically significant, the estimates suggest that the participants recruited through their neurologist may have been older, might have had a shorter disease duration and might have been less likely to receive tertiary education than the participants recruited via the other recruitment strategies. Both groups were similar in terms of sex, motor symptoms, depression, anxiety, cognition, quality of life and disease stage (Table 4).

**Table 4:** Baseline characteristics of questionnaire participants from the PRIME region who were and were not recruited through their neurologist

Variables^a^		Recruited through neurologist^b^ (n = 263)	Otherwise recruited (n = 112)	(Log–)B–weights / odds ratio [95% CI]^c^	Adj. *p*
Age: mean (SD)		71.6 (7.5)	70.4 (8.3)	-1.41 [-3.21, 0.31]	.50
Sex (men): n (%)		172 (65)	74 (66)	1.04 [0.65, 1.68]	.87
Motor symptoms: mean (SD)^d^		12.2 (7.7)	13.8 (8.1)	0.08 [-0.06, 0.22]	.66
Depression: mean (SD)^e^		11.7 (6.6)	12.5 (7.2)	0.03 [ -0.10, 0.16]	.87
Anxiety: mean (SD)		38.7 (9.3)	39.0 (9.6)	0.18 [-1.88, 2.23]	.87
Cognition: mean (SD)^f^		17.2 (3.1)	17.5 (2.9)	0.03 [-0.01, 0.07]	.57
Quality of Life: mean (SD)		73.9 (12.9)	72.4 (13.8)	-0.51 [-3.20, 2.17]	.87
Disease duration in years: mean (SD)^g^		5.36 (4.5)	6.51 (6.0)	0.13 [-0.14, 0.48]	.50
*Education: n(%)*					
Primary		101 (38)	34 (30)	Reference	
Secondary		75 (29)	32 (29)	1.31 [0.73, 2.32]	.66
Tertiary		87 (33)	46 (41)	1.62 [0.94, 2.80]	.50
Disease stage: n (%)^h^	Stage 1	77 (29)	34 (30)	Reference	
	Stage 2	91 (35)	34 (30)	0.80 [0.45, 1.45]	.73
	Stage 3	38 (14)	18 (16)	0.91 [0.43, 1.90]	.87
	Stage 4	42 (16)	15 (13)	0.66 [0.30, 1.48]	.66
	Stage 5	11 (4)	8 (7)	1.60 [0.57, 4.53]	.66

^a^SD = standard deviation; Motor symptoms*:* Movement Disorders Society Unified Parkinson Disease Rating Scale (MDS-UPDRS) Part II, ranging from 0 to 52, in which a higher score indicates a greater degree of motor symptoms. Depression: Beck Depression Inventory II (BDI), ranging from 0 to 63, in which higher scores indicate greater depressive severity. Anxiety: State Trait Anxiety Inventory for Adults (STAI); for this research, only the Trait Anxiety Scale was included, ranging from 20 to 80, in which a higher score indicates a greater degree of anxiety. Cognition: Telephone Montreal Cognitive Assessment (t-MoCA), ranging from 0 to 22, in which a higher score indicates better cognitive performance. Quality of life: Parkinson’s Disease Questionnaire-39 (PDQ-39), ranging from 0 to 100, in which a higher score indicates a better quality of life. Disease duration: Years since diagnosis of PD or parkinsonism. Education: Primary educated = no education, primary school, VMBO (see also Table S1); Secondary educated = HAVO, VWO, MBO; Tertiary educated = HBO, University, PhD. Disease stage: Hoehn & Yahr scale, this score was calculated on answers from other questionnaires, notably the UPDRS. Scores can range from 1 to 5 (disease stage 1 to 5) in which a higher stage indicates more severe disease

^b^NAs: n = 39

^c^CI = confidence interval; all continuous variables were analyzed using linear regression, all binary variables were analyzed using binomial logistic regression, all categorial variables with more than two categories, including ordinal variables, were analyzed using multinomial logistic regression. Continuous variables that did not meet the assumptions for linear regression were log transformed before conducting linear regression. All tests between recruited through neurologist and not recruited through neurologist were adjusted for age, sex and disease duration, excluding the tests for age, sex and disease duration. We reported odds ratios for all variables tested with binomial or multinomial logistic regression, log-b-weights for all log transformed variables tested with linear regression, and b-weights for all other variables tested with linear regression

^d, e, f, g^Log transformed before tested with linear regression

^h^Not 100% in total due to NAs

#### Loss to follow-up

**Fig. 3:**
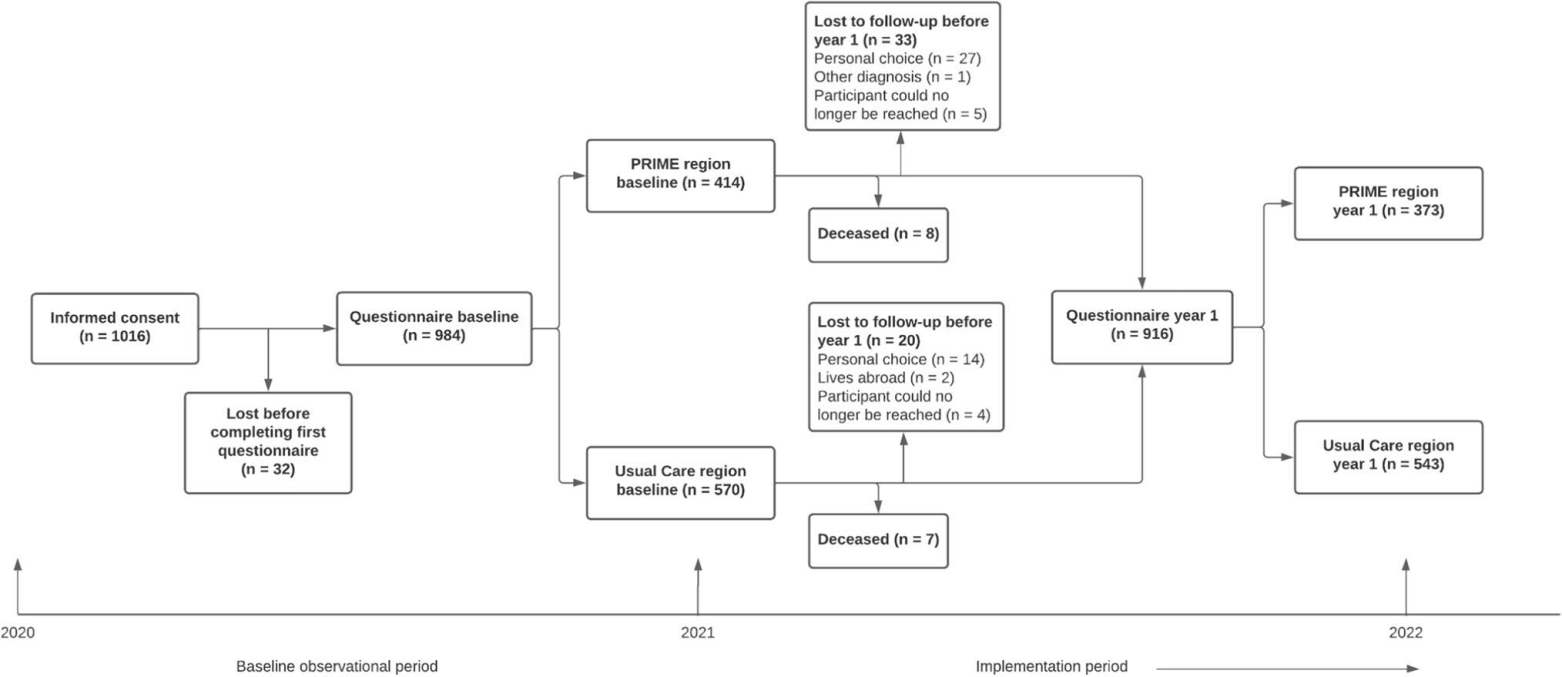
Illustration of the loss to follow-up during the first year of the PRIME-NL study

At baseline, 984 participants completed the PRIME-NL questionnaire of whom 916 (93%) were retained at the first follow-up measurement after one year (Fig. 3). 15 participants (1.5%) had deceased before the first follow-up measurement; 8 (2%) in the PRIME region and 7 (1.2%) in the UC region. 53 participants (5.4%) dropped out of the study; 33 (8.0%) in the PRIME region and 20 (3.5%) in the UC region. The most common reasons for dropping out were disease progression (40%) and the inability to reach the participant again (17%; supplementary Table 3 also displays regional data). Within the PRIME region, the participants who were lost were older and reported a poorer quality of life than those who remained. There were no other statistically significant differences between the participants who were lost and who remained in the PRIME region. Still, the PRIME region estimates suggested that the participants lost to follow-up may have had more motor and depressive symptoms, a longer disease duration, a higher disease stage and seemed less likely to receive tertiary education than the participants who remained in the study (Table 5). Within the UC region, the participants who were lost had more cognitive symptoms and reported a poorer quality of life than the participants who remained, but were comparable on all other outcomes (Table 5). Noteworthy, the differences between lost and remained participants might differ between both regions. For example, the difference in motor and depressive symptoms, as well as level of education and age, seems to be more negative for the PRIME than the UC region.

**Table 5:** Participants who were lost to follow-up compared on baseline characteristics to participants who remained in the study, separately for the PRIME and usual care region

Variables^a^		PRIME				Usual care			
		Lost to follow-up (n = 33)	Remained (n = 381)	(Log-)B-weights / odds ratio [95% CI]^b^	Adj. *p*	Lost to follow-up (n = 20)	Remained (n = 550)	(Log-)B-weights / odds ratio [95% CI]^b^	Adj. *p*
Age (years): mean (SD)		76.4 (5.4)	71.3 (7.8)	4.90 [2.16, 7.64]	.01	71.5 (7.1)	73.7 (13.1)	3.12 [-0.36, 6.60]	.21
Sex (men): n (%)		21 (64)	250 (66)	0.83 [0.40, 1.80]	.62	14 (70)	316 (58)	1.54 [0.60, 4.48]	.65
Motor symptoms: mean (SD)^c^		18.9 (11.3)	12.6 (7.7)	0.21 [-0.02, 0.44]	.14	15.2 (9.4)	12.1 (7.4)	0.06 [-0.21, 0.32]	.84
Depression: mean (SD)^d^		15.0 (6.5)	11.8 (7.0)	0.20 [-0.02, 0.41]	.16	13.4 (7.2)	11.6 (6.8)	0.11 [-0.16, 0.39]	.65
Anxiety: mean (SD)^e^		41.7 (8.7)	38.7 (9.6)	2.32 [-1.17, 5.81]	.24	41.2 (8.3)	37.3 (9.3)	4.16 [0.03, 8.30]	.18
Cognition: mean (SD)		16.4 (3.2)	17.3 (3.1)	-0.02 [-0.10, 0.06]	.62	15.4 (4.0)	18.7 (2.6)	-0.19 [-0.26, -0.13]	< .0001
Quality of Life: mean (SD)		64.2 (13.4)	73.7 (13.1)	-7.29 [-11.7, -2.89]	.01	67.1 (15.9)	74.9 (12.8)	-7.76 [-13.31, -2.21]	.04
Disease duration in years: mean (SD)^f^		6.94 (4.8)	5.57 (5.2)	0.19 [-0.05, 0.44]	.17	6.75 (5.0)	6.58 (5.3)	0.04 [-0.25, 0.33]	.82
*Education: n (%)*									
Primary		17 (52)	137 (36)	Reference		3 (15)	95 (17)	Reference	
Secondary		8 (24)	106 (28)	0.64 [0.26, 1.58]	.39	4 (20)	140 (26)	1.08 [0.23, 5.05]	.92
Tertiary		8 (24)	138 (36)	0.50 [0.20, 1.21]	.17	13 (65)	315 (57)	1.21 [0.33, 4.44]	.84
Stage of disease: n (%)^g^	Stage 1	1 (3)	114 (30)	Reference		3 (15)	164 (30)	Reference	
	Stage 2	9 (28)	125 (33)	6.49 [0.79, 52.98]	.14	3 (15)	199 (36)	0.71 [0.14, 3.63]	.84
	Stage 3	6 (18)	58 (12)	8.25 [0.93, 72.97]	.16	7 (35)	79 (14)	4.22 [1.00, 17.81]	.18
	Stage 4	10 (30)	60 (16)	11.0 [1.31, 93.69]	.10	6 (30)	80 (15)	3.67 [0.82, 16.61]	.21
	Stage 5	4 (12)	19 (5)	14.3 [1.46, 141.17]	.10	1 (5)	16 (3)	2.92 [0.26, 32.46]	.65

^a^SD = standard deviation; Motor symptoms*:* Movement Disorders Society Unified Parkinson Disease Rating Scale (MDS-UPDRS) Part II, ranging from 0 to 52, in which a higher score indicates a greater degree of motor symptoms. Depression: Beck Depression Inventory II (BDI), ranging from 0 to 63, in which higher scores indicate greater depressive severity. Anxiety: State Trait Anxiety Inventory for Adults (STAI); for this research, only the Trait Anxiety Scale was included, ranging from 20 to 80, in which a higher score indicates a greater degree of anxiety. Cognition: Telephone Montreal Cognitive Assessment (*t*-MoCA), ranging from 0 to 22, in which a higher score indicates better cognitive performance. Quality of life: Parkinson’s Disease Questionnaire-39 (PDQ-39), ranging from 0 to 100, in which a higher score indicates a better quality of life. Disease duration: Years since diagnosis of PD or parkinsonism. Education: Primary educated = no education, primary school, VMBO (see also Table S1); Secondary educated = HAVO, VWO, MBO; Tertiary educated = HBO, University, PhD. Disease stage: Hoehn & Yahr scale, this score was calculated on answers from other questionnaires, notably the UPDRS. Scores can range from 1 to 5 (disease stage 1 to 5) in which a higher stage indicates more severe disease

^b^CI = confidence interval; all continuous variables were analyzed using linear regression, all binary variables were analyzed using binomial logistic regression, all categorial variables with more than two categories, including ordinal variables, were analyzed using multinomial logistic regression. Continuous variables that did not meet the assumptions for linear regression were log transformed before conducting linear regression. All tests between lost to follow-up and remaining participants were adjusted for age, sex and disease duration, excluding the tests for age, sex and disease duration. We reported odds ratios for all variables tested with binomial or multinomial logistic regression, log-b-weights for all log transformed variables tested with linear regression, and b-weights for all other variables tested with linear regression

^c, d, e, f^Log transformed before tested with linear regression

^g^Not 100% in total due to NAs

## Discussion

The PRIME-NL study remotely evaluates the PRIME Parkinson care model, a multifaceted complex healthcare innovation. To determine both the generalizability of the findings and potential sources of bias in the questionnaire sample, we investigated whether the source population of people with PD differs between the PRIME and UC region, compared the combined questionnaire sample of participants from both regions to the source population and compared the PRIME and UC questionnaire sample on baseline characteristics and investigated the 1-year compliance. Examining similar questions for care partners and healthcare professionals was beyond the scope of this article.

### Source population differences and generalizability

According to the available healthcare claims data, people with PD in the PRIME and UC source populations were comparable at baseline regarding age, sex, comorbidities and number of fractures and pneumonia’s. Although statistically significant, the difference in disease duration between the regions is negligible. People with PD in the PRIME region had a somewhat lower socioeconomic status and fewer PRIME participants used medications for anxiety, depression and cognitive symptoms. Furthermore, the comparison of CBS data between the PRIME and UC region showed no meaningful differences between the regions. Naturally, the interpretability of the CBS data is somewhat limited as the database is not PD-specific.

Given that the source populations were highly similar, we assessed the generalizability of the questionnaire sample by comparing the combined questionnaire sample of participants from both regions to the entire source population. However, only four variables were measured in both the healthcare claims database and the questionnaire sample, limiting the breadth of our comparison. The questionnaire sample shows a slight underrepresentation of older people with PD compared to the source population. Compared to other prospective longitudinal cohort studies, the PRIME-NL questionnaire participants are indeed younger when we correct for disease duration (PRIME-NL age at diagnosis = 61.8 years, ParkWest, Oxford Discovery and CaMpaIGN range = 66.1 – 70.2 years) [13–15]. This underrepresentation of elderly is not uncommon in research studies [16] and can be explained by a multitude of factors such as disease progression, cognitive state and comorbidities. Specifically, our recruitment methods typically required technological skills such as visiting a website or active engagement in the community such as attending a conference, which might be easier for younger people and people who are less affected by parkinsonism. However, since the underrepresentation of older people with PD in the questionnaire sample is only modest, we do not think this forms a substantial limitation in generalising the eventual results of the PRIME-NL study to the broader population of people with PD. Besides the difference in age, the questionnaire sample also had a slightly longer disease duration which could partially be explained by a delay of the clinical diagnosis registration in the healthcare claims data. Finally, the questionnaire sample participants were more likely to experience a pneumonia, which could partially be due to their longer disease duration.

For future studies, we recommend to put extensive effort into recruiting people personally, both offline and online, to reach the full spectrum of the parkinsonism population. Besides our own positive recruitment experiences, another study demonstrated that one or more telephone calls recruited an additional 31% of participants who differed on several characteristics, compared to those without phone contact, such as being more frail [17]. Furthermore, online advertisements through social media platforms can be used to successfully reach underrepresented groups, including geographically distant and late stage people with PD [18]. Our study would have benefited from such additional recruitment strategies, as the questionnaire sample lacks the inclusion of people with parkinsonism with a migratory background and with a primary and secondary educational attainment (Table 2B and Table 3).

### Selection and confounding bias in the questionnaire sample

#### Baseline characteristics and recruitment strategy

Participants in the questionnaire sample in the PRIME region were older than the participants in the UC region and were also more affected by their parkinsonism given their worse cognition, anxiety, quality of life and higher BMI (although the latter three require careful interpretation). These differences highlight the presence of selection bias, given that the source populations were similar or showed a reversed effect, e.g., more anxiety medication in the UC source population. We hypothesized that the selection bias might have been caused by the recruitment letter from the neurologist in the PRIME region. A letter, sent by the participants own treating neurologist, is more personal and could have reached older and more affected people who might well be missed by general recruitment methods. The general recruitment methods required more digital skills, which might explain why participants in the UC region were younger and completed the questionnaire more frequently online rather than on paper compared to the PRIME participants. Indeed, participants in the PRIME region recruited via their neurologist seemed to be older and less likely to receive tertiary education than participants recruited via other recruitment strategies, although they also might have had a shorter disease duration. Note that these differences were not statistically significant, so we could not find strong evidence for our hypothesis that the letter reached a specific subgroup of people with PD, resulting in the selection bias we found. However, we have identified two alternative explanations. First, the letter has reached a subgroup but our data on recruitment method are misleading. Some PRIME participants reported to be recruited by their neurologist but had entered the study before the recruitment letters were sent out. Also, some UC participants had indicated that they had been recruited by their neurologist despite not receiving a letter, maybe because their neurologist mentioned the study during a clinical visit. We have attempted to correct such cases before the analyses, but incorrect recruitment method classifications might reside disproportionally more in the PRIME region, leading to differential measurement error and thereby information bias. Second, the beneficial effect of sending a recruitment letter on recruiting specific subgroups might be limited. For example, participants living in the PRIME region live closer to the research centre from which PRIME-NL is coordinated (Radboud university medical center), which may already create a stronger sense of involvement and readiness to participate for this ‘local’ initiative, reducing the additive effect of the letter.

The UC rather than the PRIME questionnaire sample seems to be diverging from the source population. For example, 58% of the participants in the UC region received tertiary education, against 35% of the participants in the PRIME region. According to the CBS data, both regions should have approximately 30% tertiary educated people, assuming that no major association between education and PD is known [10]. Furthermore, the average age of the PRIME questionnaire participants (71.8) is closer to the average age of their source population (72.9) than is the case for the UC region (sample 68.2, source 72.7). We have oversampled in the PRIME region since this region is much smaller (n = 1430) than the UC region (n = 26,250). Relatively more people with PD from the PRIME region were included, making them a better representation of their source population.

We did not investigate potential sources of information bias, such as the COVID-19 pandemic or being unblinded to the study group. For example, COVID-19 could have differentially affected the PRIME or the UC region over the baseline year, regionally and temporally reducing well-being. The unblinding of participants might occur after the first innovations have been implemented. Once participants are unblinded, information bias could arise during follow-up as people in the PRIME region might answer more positively since they are aware of the additional care they are receiving.

#### Loss to follow-up

We retained 93% of participants after the first year of follow-up (94.6% when excluding deceased participants). This compliance percentage is remarkably high, although we are not aware of similar longitudinal healthcare model evaluations within and beyond the field of parkinson(ism) to compare our findings to. As an example, the ParkWest cohort study achieved a 1-year compliance of 98.4% [15], but investigated disease progression and therefore only recruited newly diagnosed people with PD. The PRIME-NL questionnaire participants have a higher average disease duration which is typically associated with more motor and cognitive impairments hampering research participation. We assume that these impairments also make it more difficult for people with parkinson(ism) to be retained in longitudinal research when compared to other chronic conditions such as diabetes mellitus and chronic obstructive pulmonary disease.

The retention of participants in our study is most likely due to a comprehensive series of activities developed by the assessment team for the present and also other studies [19]. These activities had been devised together with several participants to match their needs more closely and include an annual personal contact moment over telephone, newsletters with research updates, a Christmas card and the presence of a helpdesk during office hours to answer questions [19]. Despite these activities being equally implemented for both regions, we lost more questionnaire sample participants in the PRIME than in the UC region after the first year. A logical explanation would be that the PRIME participants on average were older and more affected by parkinsonism at baseline, i.e., they experienced more anxiety, cognitive impairments, and a lower quality of life. These factors increase the likelihood that people with parkinsonism will reach a stage in their disease in which they are no longer willing to complete the questionnaires. This hypothesis is also supported by our data, as the most frequently reported reason to resign from participation was disease progression. Furthermore, outcomes related to disease burden were associated with reduced compliance, including motor and cognitive symptoms and quality of life. Although we lacked power to conduct statistical tests for interaction, the PRIME region seems to have suffered more from selective attrition, i.e., more affected participants were lost compared to the UC region. Future evaluation of the participants lost to follow-up is necessary, as power might become sufficient to perform statistical tests in later years of PRIME-NL.

In conclusion, the PRIME and UC source populations are highly comparable and the questionnaire sample participants are a reasonable representation of the source populations. These findings support the generalizability of the PRIME-NL evaluation for people with PD. However, the evaluation of the questionnaire sample data can be affected in various ways. On the one hand, the selection bias introduced at baseline led to the inclusion of older and more affected participants in the PRIME region. This selection bias could become a source of confounding as age and disease progression negatively predict several outcomes. Even when we correct for baseline differences in the final evaluation, the impact of PRIME Parkinson care could be underestimated due to increased disease progression, less room for improvement in healthcare (ceiling effect) or difficulties in reaching the participants in the PRIME region. On the other hand, selective attrition of more affected participants in the PRIME region could result in overestimating the positive effect of PRIME Parkinson care (Fig. 1). We will explore various statistical methods to account for these differences, for example through inverse probability weighting or propensity score matching. Ultimately, this study brings us closer to the final purpose of PRIME-NL: to evaluate whether PRIME Parkinson care can improve care for all people with parkinsonism.

### Supplementary Information

Below is the link to the electronic supplementary material.

Supplementary file1 (DOCX 47 kb)

## References

[1] Dorsey ER, Sherer T, Okun MS, Bloem BR. The emerging evidence of the Parkinson pandemic. J Parkinsons Dis. 2018;8:S3-8. 30584159 10.3233/JPD-181474 PMC6311367

[2] Bloem BR, Okun MS, Klein C. Parkinson’s disease. The Lancet. 2021;397:2284–303. 10.1016/S0140-6736(21)00218-X 33848468

[3] Bloem BR, Henderson EJ, Dorsey ER, Okun MS, Okubadejo N, Chan P, et al. Integrated and patient-centred management of Parkinson’s disease: a network model for reshaping chronic neurological care. The Lancet Neurology. 2020;19:623–34. 32464101 10.1016/S1474-4422(20)30064-8 PMC9671491

[4] Tenison E, Smink A, Redwood S, Darweesh S, Cottle H, Van Halteren A, et al. Proactive and integrated management and empowerment in parkinson’s disease: designing a new model of care. Parkinson’s Disease. 2020;2020:1–11. 10.1155/2020/8673087 PMC7149455 32318261

[5] Bodenheimer T, Sinsky C. From triple to quadruple aim: care of the patient requires care of the provider. Ann Fam Med. 2014;12:573–6. 25384822 10.1370/afm.1713 PMC4226781

[6] Berwick DM, Nolan TW, Whittington J. The triple aim: care, health, and cost. Health Aff (Millwood). 2008;27:759–69. 18474969 10.1377/hlthaff.27.3.759

[7] Ypinga JHL, Van Halteren AD, Henderson EJ, Bloem BR, Smink AJ, Tenison E, et al. Rationale and design to evaluate the PRIME Parkinson care model: a prospective observational evaluation of proactive, integrated and patient-centred Parkinson care in The Netherlands (PRIME-NL). BMC Neurol. 2021;21:286. 34294077 10.1186/s12883-021-02308-3 PMC8298196

[8] Lithander FE, Tenison E, Ypinga J, Halteren A, Smith MD, Lloyd K, et al. Proactive and integrated management and empowerment in parkinson’s disease protocol for a randomised controlled trial (PRIME-UK) to evaluate a new model of care. Trials. 2023;24:147. 36849987 10.1186/s13063-023-07084-8 PMC9969590

[9] Centraal Bureau voor de Statistiek (2023). StatLine electronic database [Internet]. Accessed on 14 July 2023. Available from: https://opendata.cbs.nl/statline/#/CBS/nl/

[10] Ben-Shlomo Y, Darweesh SKL, Llibre-Guerra J, Marras C, San Luciano M, Tanner CM. The epidemiology of Parkinson’s disease. The Lancet. 2024;403:283-292. 10.1016/S0140-6736(23)01419-8 PMC11123577 38245248

[11] Benjamini Y, Hochberg Y. Controlling the false discovery rate: a practical and powerful approach to multiple testing. J Roy Stat Soc: Ser B (Methodol). 1995;57:289–300. 10.1111/j.2517-6161.1995.tb02031.x

[12] R Core Team (2023). R: A language and environment for statistical computing. R Foundation for Statistical Computing, Vienna, Austria. URL https://www.R-project.org/.

[13] Szewczyk-Krolikowski K, Tomlinson P, Nithi K, Wade-Martins R, Talbot K, Ben-Shlomo Y, et al. The influence of age and gender on motor and non-motor features of early Parkinson’s disease: Initial findings from the Oxford Parkinson Disease Center (OPDC) discovery cohort. Parkinsonism Relat Disord. 2014;20:99–105. 24183678 10.1016/j.parkreldis.2013.09.025

[14] Williams-Gray CH, Mason SL, Evans JR, Foltynie T, Brayne C, Robbins TW, et al. The CamPaIGN study of Parkinson’s disease: 10-year outlook in an incident population-based cohort. J Neurol Neurosurg Psychiatry. 2013;84:1258–64. 23781007 10.1136/jnnp-2013-305277

[15] Bjornestad A, Pedersen KF, Tysnes O-B, Alves G. Clinical milestones in Parkinson’s disease: a 7-year population-based incident cohort study. Parkinsonism Relat Disord. 2017;42:28–33. 28578818 10.1016/j.parkreldis.2017.05.025

[16] Maas BR, Bloem BR, Ben-Shlomo Y, Evers LJW, Helmich RC, Kalf JG, et al. Time trends in demographic characteristics of participants and outcome measures in Parkinson’s disease research: a 19-year single-center experience. Clin Park Relat Disord. 2023;8: 100185. 36793589 10.1016/j.prdoa.2023.100185 PMC9923175

[17] Tenison E. Capturing complexity, comorbidity and frailty in people with parkinsonism and understanding their impact. PhD thesis University of Bristol; 2023.

[18] Dobkin RD, Amondikar N, Kopil C, Caspell-Garcia C, Brown E, Chahine LM, et al. Innovative recruitment strategies to increase diversity of participation in Parkinson’s disease research: the fox Insight cohort experience. J Parkinsons Dis. 2020;10:665–75. 32250321 10.3233/JPD-191901 PMC7242847

[19] Meinders MJ, Marks WJ, van Zundert SBM, Kapur R, Bloem BR. Enhancing participant engagement in clinical studies: strategies applied in the personalized Parkinson project. J Parkinsons Dis. 2023;13:637–40. 37092234 10.3233/JPD-225015 PMC10357143

