# Supplementary material for: Assessing the validity of a Parkinson’s care evaluation: the PRIME-NL study
Source: Eur J Epidemiol. 2024 May 30;39(7):811–25. doi: 10.1007/s10654-024-01123-7 (PMC11343810; doi:10.1007/s10654-024-01123-7)
Supplement: Supplementary file 1 — Supplementary file1 (DOCX 47 kb) [file 10654_2024_1123_MOESM1_ESM.docx]

Supplement

**Table 1: Overview on operationalization of the variables.**

| **Measuring tool** | **Variable** | **Explanation** | **Data processing** |
| --- | --- | --- | --- |
|  | Region | PRIME or Usual Care region |  |
| General PRIME questionnaire | Sex | Man, woman, other |  |
|  | Age | In years |  |
|  | Diagnosis | Diagnoses were verified via a letter from the general practitioner or neurologist. | Created two groups: people with Parkinson’s disease or with atypical parkinsonism |
|  | Disease duration | In questionnaire: Participants entered age of diagnosis.  In healthcare claims data: the number of years from first DBC 501 code. |  |
|  | Migratory background | Participants could indicate which ethnic group they considered themselves to belong to. Options: Dutch, Turkish, Moroccan, Surinamese, Antillean / Aruban, other. | Recoded to 1 = Dutch, 2 = other. If a combination of Dutch and another ethnicity was reported, then this was scored as ‘other’. |
|  | Recruitment procedure | Options: via my neurologist, via ParkinsonNEXT, via the Parkinson Association, via family/friends, via the person with parkinsonism I am taking care of, via other carers, via social media, via ParkinsonNet, other. | Recoded to two options: via my neurologist and not via my neurologist. Few people chose ‘other’ and gave an explanation as ‘a letter from the hospital’; this was recoded to via my neurologist. |
|  | Education  (diploma obtained) | Options^a^: no education, primary school, VMBO, HAVO, VWO, MBO, HBO, University, PhD, other. | Three groups^b^:  1= primary educated (no education, primary school, VMBO)  2= secondary educated (HAVO, VWO, MBO),  3= tertiary educated (HBO, University, PhD) Participants explained ‘other’ in the questionnaire, and this was recoded to one of the education levels. |
|  | Living situation | Options: alone, with partner, with partner and children, in an institution, assisted living^c^, sheltered living^d^, living with another family member than partner. |  |
|  | Work situation | Options: fulltime, parttime, self-employed, education following not paid by employer, retired, unemployed, incapacitated, active in household caring for children or other people, unemployed and receiving sickness benefit, voluntary work. |  |
|  | Smoking | Contains two questions: currently smoking? (Yes/no) Smoked in the past? (Yes/no). | Recoded to 0 = never, 1 = in the past, 2 = current smoker. |
|  | Alcohol consumption | Options: never, very rarely on special occasions, occasionally, on less than 5 days a week, on 5 or more days a week. |  |
|  | BMI | Body Mass Index calculated from length and weight from questionnaire. | BMI = weight in kg / (length in centimetres/100)^2^ . |
|  | Comorbidities: 5 unique variables | Cardiovascular disease, pulmonary disease, musculoskeletal disorder, endocrine or metabolic disorder, neuropsychiatric disorder, cancer, none of the above. | 0= I don’t have a disease in this category, 1= I do have a disease in this category. |
|  | Complications: 4 unique variables | Urinary tract infection, pneumonia, falling, neuropsychiatric disorders (hallucinations) in the past year: recoded to not reported, reported, reported and led to hospital admission. | Recoded to 0= not had, 1= had, 2= had and led to hospital admission. |
| Parkinson’s Disease Questionnaire-39 (PDQ-39)^1^ | Quality of Life | Contains questions in eight domains: mobility, general daily living tasks, emotional, stigma, support, cognition, communication, discomfort. | The total scores of all domains were summed up and averaged. Scores can range from 0 to 100, in which zero is the best and 100 the worst quality of life. The values were recoded into a reversed scale. |
| Beck Depression Inventory II (BDI)^2^ | Depression | The score of the BDI reflects the intensity of depression. Scores from 0 through 9 indicate no or minimal depression, scores from 10 through 18 indicate mild to moderate depression, scores from 19 through 29 indicate moderate to severe depression, scores from 30 through 63 indicate severe depression. | Questions can be answered with ratings from 0 to 3 and are summed, with a total maximum score of 63, in which higher scores indicate greater depressive severity. We used the total score for analysis. |
| State Trait Anxiety Inventory for Adults (STAI)^3^ | Anxiety | There are 2 subscales within this measure. The State Anxiety Scale evaluates the current state of anxiety, the Trait Anxiety Scale evaluates relatively stable aspects of “anxiety proneness,” including general states of calmness, confidence, and security. For this research, only the Trait Anxiety Scale was included as we are interested in the anxiety in general, not during the specific moment of filling in the questionnaire. | Scores can range from a minimum of 20 to a maximum of 80 in a linear scale, in which a higher score indicates a greater degree of anxiety. We used the total score of trait anxiety for analysis. |
| Movement Disorders Society Unified Parkinson Disease Rating Scale (MDS-UPDRS): Part II^4^ | Motor symptoms | Only part II of the complete questionnaire is used. These questions address the extent to which a person is limited in daily activities as a result of motor symptoms. | Scores can range from 0 to 52, in which a higher score indicates a greater degree of motor symptoms. We used the total score for analysis. |
| Telephone Montreal Cognitive Assessment (t-MoCA)^5^ | Cognitive performance | Several cognitive tasks are performed through a telephone interview. The item concerning location (place and city) could not be verified via telephone and was therefore not asked. Every participant got +2 on their score for this missing question. Furthermore, the day of the week was not asked for by some of the participants. These participants got +1 on their score to correct for this missing question. | Scores can range from 0 to 22, in which a higher score indicates better cognitive performance. We used the total score for analysis. |
| Hoehn & Yahr | Stage of disease | The Hoehn and Yahr scale is a system to describe in what stage of Parkinson’s disease a person is. Scores can range from 1 to 5 (disease stage 1 to 5) in which a higher stage indicates more severe disease. | We calculated H&Y based on answers from other questionnaires notably the UPDRS, e.g., for the presence of bilateral impairments or mobility aids. 1= Unilateral involvement only, 2= Bilateral involvement without impairment of balance, 3= Mild to moderate bilateral disease; some postural instability; physically independent, 4= Severe disability; still able to walk or stand unassisted, 5= Wheelchair bound or bedridden unless aided. |
| COVID-19 questionnaire | COVID-19 burden | This questionnaire contains 8 questions about the personal social impact of COVID-19, as well as the impact participants have experienced on access to healthcare. Every question could be answered with a scale from 0 (not experienced) to 5 (often experienced). | The mean sum of scores is used (sum / number of questions answered), ranging from 0 to 5, in which a higher score indicates a higher COVID-19 burden. |

^a^VMBO, HAVO and VWO are types of education during high school, where VMBO is re-vocational secondary education, HAVO is senior general secondary education and VWO is pre-university education. MBO is secondary vocational education training, HBO is higher professional education.

^b^Recoded based on how CBS (Central Bureau of Statistics) classifies education in the Netherlands.

^c^Independently living and receiving outpatient support from a housing or welfare organisation.

^d^Living in a house belonging to a housing or welfare organisation.

**Supplementary table 2: Mode of data collection**

| Mode of data collection | PRIME  (n = 414) | Usual care  (n = 570) |
| --- | --- | --- |
| Online: n (%) | 222 (54) | 447 (78) |
| Paper: n (%) | 187 (45) | 119 (21) |
| Telephone: n (%) | 5 (1.2) | 4 (0.7) |

**Supplementary table 3: Reasons for dropping out of the study.**

| Reason: n (%) | PRIME  (n = 33) | Usual care  (n = 20) | Total  (n = 53) |
| --- | --- | --- | --- |
| Personal choice | 27 (82) | 14 (70) | 41 (77) |
| - Disease progression - Too intensive - Too confrontational - Questions do not match disease - Other / unknown | 14 (42)  5 (15)  2 (6)  1 (3)  5 (15) | 7 (35)  1 (5)  3 (15)  1 (5)  2 (10) | 21 (40)  6 (11)  5 (9)  2 (4)  7 (13) |
| Other diagnosis | 1 (3) | 0 (0) | 1 (2) |
| Lives abroad | 0 (0) | 2 (10) | 2 (4) |
| Participant can no longer be reached | 5 (15) | 4 (20) | 9 (17) |

**Supplementary file S1: Information on the extraction and processing of data on the general population from the Central Bureau of Statistics (CBS)**

We extracted data on a provincial level, coding the province Noord-Brabant, Gelderland and Limburg as the PRIME region and the other nine provinces as the UC. We gathered the data from the StatLine database of the CBS, weighing all outcomes based on the number of inhabitants of each province where necessary. Where possible, we collected data of people >60 years to best resemble our PD population as well as data as close to the year 2020 which was the PRIME-NL baseline year.

We gathered the **number of people** in the UC and PRIME region from the official registry of citizens of the Netherlands. These data were used when the CBS database only provided percentages. When we had access to more precise estimates of the total group size, we used those, e.g. migratory background. We selected the average amount of people across the year, classified by age depending on which age group and database year was most appropriate:

<https://opendata.cbs.nl/#/CBS/nl/dataset/03759ned/table?dl=39E0B>

For **migratory background** we used the variable ‘herkomstland’, with the country of origin defined as outside the Netherlands when the person or someone’s parents are not born in the Netherlands. Closest data was only available of the year 2022. We used the number of people provided by this specific table: PRIME n = 1,547,290; UC n = 2,649,563

<https://opendata.cbs.nl/statline/#/CBS/nl/dataset/85458NED/table?ts=1689069161114>

For **BMI**, we used overweight (BMI >= 25) as a replacement because BMI was not available. We used the data of people >65 years because >60 was unavailable; data is from the 2022 Gezondheidsmonitor. This dataset also includes current **smoking** behaviour and people who consume more **alcohol** than regular (>21 drinks for men and >14 drinks for women). Here, we used the number of people calculated in the first step: PRIME n = 1,174,546; UC n = 2,016,678

<https://opendata.cbs.nl/statline/#/CBS/nl/dataset/85563NED/table?ts=1687260675027>

For **COVID-19**, we used the registry of hospitalizations from 2020, specifically for age >65. We used the number of people calculated in the first step: PRIME n = 1,262,229; UC n = 2,268,434

<https://opendata.cbs.nl/statline/#/CBS/nl/dataset/84523NED/table?ts=1687265201092>

For **education**, no provincial data was accessible for people >60. We had to resort to data from 2018 of people >18 years. Therefore, we used the number of people calculated in the first step: PRIME n = 4,620,529 ; UC n = 9,135,511

[Percentage mensen in opleidingsniveau en leeftijdgroep (cbs.nl)](https://www.cbs.nl/nl-nl/maatwerk/2019/19/percentage-mensen-in-opleidingsniveau-en-leeftijdgroep)

For **living** **situation**, we classified people either as living alone or with someone, i.e. partner or children. We used the 2020 data for all people >60. Since this concerned household data, we used the n provided by this specific topic’s table: PRIME n = 1,029,544; UC n = 1,923,901

[StatLine - Huishoudens; samenstelling, grootte, regio, 1 januari (cbs.nl)](https://opendata.cbs.nl/#/CBS/nl/dataset/71486ned/table?ts=1689166648244)

**References**

1. Peto V, Jenkinson C, Fitzpatrick R, Greenhall R. The development and validation of a short measure of functioning and well being for individuals with Parkinson’s disease. *Quality of Life Research*. 1995;4(3):241-248. doi:10.1007/BF02260863

2. Beck AT, Ward CH, Mendelson M, Mock J, Erbaugh J. An inventory for measuring depression. *Arch Gen Psychiatry*. 1961;4(6):561. doi:10.1001/archpsyc.1961.01710120031004

3. Spielberger CD, Gorsuch RL, Lushene R, Vagg PR, Jacobs GA. *Manual for the State-Trait Anxiety Inventory.* Consulting Psychologists Press; 1983.

4. Goetz CG, Tilley BC, Shaftman SR, et al. Movement Disorder Society-Sponsored Revision of the Unified Parkinson’s Disease Rating Scale (MDS-UPDRS): Scale presentation and clinimetric testing results. *Movement Disorders*. 2008;23(15):2129-2170. doi:10.1002/mds.22340

5. Pendlebury ST, Welch SJV, Cuthbertson FC, Mariz J, Mehta Z, Rothwell PM. Telephone assessment of cognition after transient ischemic attack and stroke. *Stroke*. 2013;44(1):227-229. doi:10.1161/STROKEAHA.112.673384
